# Phase-field modeling of border cell cluster migration in *Drosophila*

**DOI:** 10.1371/journal.pcbi.1014176

**Published:** 2026-04-22

**Authors:** Naghmeh Akhavan, Alexander George, Michelle Starz-Gaiano, Bradford E. Peercy

**Affiliations:** 1 Department of Mathematics and Statistics, University of Maryland Baltimore County, Baltimore, Maryland, United States of America; 2 Department of Biological Sciences, University of Maryland Baltimore County, Baltimore, Maryland, United States of America; University of Utah, UNITED STATES OF AMERICA

## Abstract

Collective cell migration is a fundamental biological process that drives events such as embryonic development, wound healing, and cancer metastasis. In this study, we develop a biophysically informed phase-field model to investigate the collective migration of the border cell cluster in the *Drosophila melanogaster* egg chamber. Our model captures key aspects of the egg chamber architecture, including the oocyte, nurse cells, and surrounding epithelium, and incorporates both mechanical forces and biochemical cues that guide cell migration.We introduce the Tangential Interface Migration (TIM) force which captures contact-mediated propulsion generated along interfaces between the border cell cluster and surrounding nurse cells. Our simulations reveal three key features of TIM-driven migration that distinguish it from previous forms of chemotaxis: (1) the explicit nature of border cell–nurse cell overlap to initiate movement (*i.e.*, border cells cannot move without a nurse cell substrate), (2) motion is tangential to border cell-nurse cell interfaces, and (3) persistent migration occurs even in regions where the spatial slope of chemoattractant is decreasing. Additionally, we demonstrate that with or without geometry-mediated alterations in chemoattractant distribution such as at intercellular junctions, we can vary induced migration pauses, independent of mechanical confinement. We capture an experimentally observed transition to dorsal migration at the oocyte with a sustained medio-lateral chemical cue of small amplitude. The results show how spatial constraints and interfacial forces shape collective cell movement and highlight the utility of phase-field models in capturing the interplay between tissue geometry, contact forces, and chemical signaling.

## Author summary

Collective cell migration plays a central role in development, immune response, and disease progression. Understanding how migrating cell groups interpret environmental cues to coordinate movement is essential for uncovering the principles relating tissue organization and invasion. Using a phase-field modeling framework, this study investigates the collective migration of the border cell cluster during *Drosophila* (fruit fly) oogenesis. The model integrates the geometry of the egg chamber and spatial configuration of small extracellular spaces, distribution of chemoattractants, receptor-mediated signaling, and a newly proposed Tangential Interface Migration (TIM) force. The TIM force captures a form of contact-mediated propulsion, requiring the cluster to engage with surrounding nurse cells to generate tangential traction at interfaces, mimicking biological behaviors such as sliding and crawling observed in live imaging studies. Our findings reveal how chemical and mechanical guidance mechanisms jointly contribute to directed migration, and demonstrate that TIM forces can effectively capture chemotactic forces by integrating both mechanical and spatial guidance features.

## Introduction

Cell migration is a fundamental biological process that underlies diverse phenomena such as embryonic morphogenesis, wound healing, immune surveillance, and cancer metastasis [1–5]. In contrast to single-cell motility [2,6–13], collective migration involves groups of cells that maintain intercellular junctions and coordinate their movement in response to both internal signaling and external environmental cues [3,4,14–17]. Unraveling the principles that govern such coordinated behaviors is crucial not only for understanding normal tissue development but also for identifying how dysregulated migration contributes to pathological conditions, including tumor invasion and metastatic dissemination.

**Fig 1:**
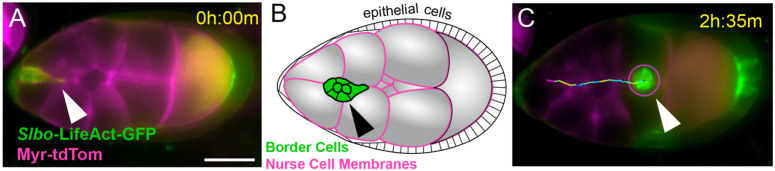
Spatio-Temporal Progression of the Border Cell Cluster. **(A, C)** Live imaging of border cell cluster migration in the Drosophila egg chamber. Time-lapse images showing the migration of the border cell cluster (green, Slbo-LifeAct-GFP) through the nurse cell complex toward the oocyte at the right. The cell membranes are labeled with Myr-tdTomato (magenta) expressed under the control of the matα promoter. The white arrowhead indicates the position of the border cell cluster at the time shown at the top right (hours:min). In **(C)**, the border cells have reached the oocyte. **(B)** A cartoon of the egg chamber when border cells (indicated with black arrowhead) have migrated part of the way, with cell types labeled.

The *Drosophila melanogaster* egg chamber is an organized structure that serves as a powerful model for studying collective cell migration [15,16]. Each egg chamber consists of 16 interconnected germline cells - 15 nurse cells and one oocyte - surrounded by a somatic epithelium of follicle cells (Fig 1). The follicle cells, nurse cells and the oocyte play distinct roles in oogenesis within the egg chamber [18–20]. At the anterior and posterior pole (ends) of the egg chamber are small groups of specialized somatic cells called polar cells, which play a role in signaling and organizing cell migration. A subset of anterior follicle cells is specified as the border cell cluster, which consists of 6–8 migratory epithelial cells that include two non-migratory polar cells and 4–6 motile border cells. During stages 8 and 9 of oogenesis, the border cell cluster detaches from the anterior epithelium and migrates collectively between the large, immobile nurse cells toward the posterior of the egg chamber, ultimately reaching the oocyte [15,16,18]. Border cell migration is guided by tissue topography [21,22] and presumed spatial gradients of chemoattractants, including PVF1, a Platelet Derived Growth Factor and Vascular Endothelial Growth Factor (PDGF/VEGF) family ligand and Epidermal Growth Factor (EGF)-like ligands secreted from the oocyte [21,23–27]. This guidance occurs through receptor-ligand interactions involving receptor tyrosine kinases (RTKs) expressed on the surface of border cells, which activate intracellular signaling pathways that promote polarized protrusion and directional motility [15,23,24,26,28–33]. As demonstrated by George et al. [27], the effectiveness of this chemotactic response is shaped by tissue geometry, which alters local chemoattractant distribution and modulates migration speed along the path to the oocyte. The physical environment through which the cluster travels is highly constrained, involving complex geometry, deformable neighboring cells, and narrow heterogeneous extracellular spaces between cells that can modulate signal distribution and mechanical resistance.

Mathematical models have been instrumental in exploring the mechanisms of collective cell migration. Traditional approaches include agent-based models (ABMs), vertex models, and cellular automata [34–39], which offer discrete representations of individual cell behaviors. While powerful in capturing rule-based cell interactions, these methods face limitations when addressing continuous and dynamic shape changes, topological rearrangements, and spatially distributed biochemical fields. In particular, they often require explicit interface tracking or introduce geometric artifacts that hinder the biophysically appropriate simulation of deformation and coordinated group migration within tightly packed tissues.

To address these challenges, we adopt a continuum-based phase-field modeling framework. Phase-field methods represent individual cells as continuous scalar fields, allowing for seamless simulation of complex morphological changes, interface dynamics, and collective rearrangements without explicit tracking of cell boundaries [40–46]. This approach is especially well-suited to multicellular systems with mechanical and chemical couplings, where cells interact through adhesion, volume exclusion, and signaling gradients within a dynamic microenvironment. Although phase-field models have been increasingly applied to biological systems [38,41,47–51], many existing studies simplify key aspects of the tissue architecture. For example, the spatial heterogeneity of the extracellular space is often overlooked or homogenized, rather than explicitly represented in the model. Moreover, cell-to-cell variation in size, adhesion properties, and receptor-mediated signaling dynamics is frequently omitted. These simplifications can obscure the complex interplay between tissue geometry, biochemical gradients, and interfacial forces that jointly shape directed collective migration.

In this study, we develop a biologically informed, multi-cellular phase-field model of border cell cluster migration in the *Drosophila* egg chamber. Our model introduces two innovations: a biophysically grounded tangential interface migration (TIM) force that models contact-mediated propulsion along cluster–nurse cell interfaces, enabling migration even in shallow or irregular chemoattractant gradients. Each cell type in the system (nurse cells, the oocyte, and the border cell cluster) is represented by a separate phase-field variable. The full model is governed by a system of partial differential equations derived from a biophysically motivated energy functional incorporating volume preservation, interfacial tension, and cell-cell adhesion.

## Methods and results

We followed the protocol for culturing *Drosophila melanogaster* stage 9 egg chambers for live imaging of [27,52] for the genotypes indicated. We utilize the multi-cellular phase-field method inspired by [41,45] to model collective cell migration in the *Drosophila* egg chamber.

### Phase field model of egg chamber architecture

**Fig 2:**
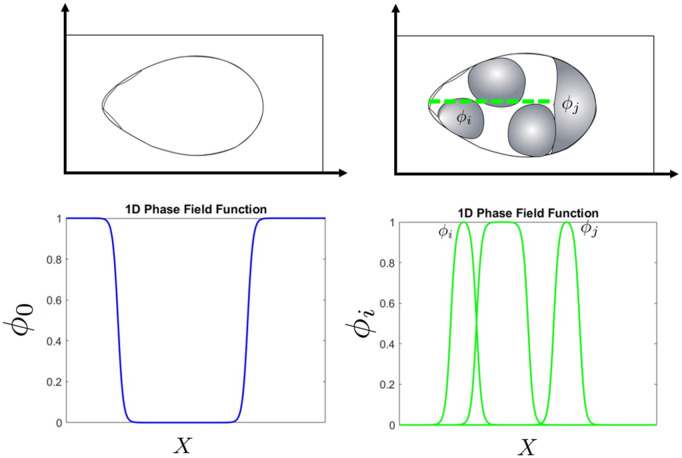
Schematic representation of the phase-field model. (Left column) The epithelial layer of the egg chamber is considered inside a rectangular region. The egg chamber is described by $\phi_{0}$ (blue). We consider the inside $\phi_{0}=0$ and outside $\phi_{0}=1$ with the transition region between them as effectively the epithelial layer. (Right column) The gray regions are indicated to nurse cells and oocyte and are considered as phase field $\phi_{i}$’s. Cross sectional line is taken through the egg chamber (green dashed). 1D phase-field representation of cells along the corresponding cross-section.

We model the egg chamber and the cells within it using a phase-field energy framework. The system includes phase variables for the epithelial boundary $\phi_{0}(x,t)$, the nurse cells $\phi_{m}(x,t)$ with indices m=1,…,M=6, the border-cell cluster $\phi_{c}(x,t)$, and the oocyte $\phi_{oct}(x,t)$. Here, $x\in \Omega \subset {\mathbb{R}}^{n}$ denotes the spatial domain (*n* = 2 in this study; see the top panel of Fig 2), and *t* ≥ 0 denotes time.

For simplicity, the entire border-cell cluster is represented by a single phase variable $\phi_{c}(x,t)$ rather than modeling individual border cells. Each phase variable $\phi_{j}$ represents a distinct cellular domain, where $\phi_{j}=1$ denotes the interior of the corresponding cell and $\phi_{j}=0$ its exterior. The epithelial phase variable $\phi_{0}$ defines the egg chamber boundary, with $\phi_{0}=0$ inside the egg chamber and $\phi_{0}=1$ outside (Fig 2).

Throughout this work, lower-case indices *m* are reserved exclusively for nurse cells. When generic summations over all cellular domains are required, we use capital indices, with the total number of cell domains given by *N* = 8 (six nurse cells in a cross section of the egg chamber, the border-cell cluster, and the oocyte).

In our model, individual cells are represented by phase-field variables that are subject to constraints enforcing volume preservation and structural integrity including during border cell migration through the egg chamber. The evolution of each phase-field is governed by a Ginzburg–Landau-type equation [53,54], derived as a gradient flow of the system’s free energy functional.

For simplicity of the model, we consider $\phi_{m}$ for the border cell cluster, nurse cells, and oocyte (*i.e.*, number of cells, *N* = 8). We consider the free energy functions of cells as follows:


$$E_{0}=\sum_{m=1}^{N}\int \left[\frac{\epsilon_{m}^{2}}{2}|\nabla \phi_{m}{|}^{2}+g(\phi_{m})\right]dx+\alpha_{m}\sum_{m=1}^{N}(V_{m}(t)-{\bar{V}}_{m}(t){)}^{2},$$ (1)

where $g(\phi_{m})=\frac{1}{4}\phi_{m}^{2}(1-\phi_{m}{)}^{2}$ is a double-well function in Ginzburg-Landau equation and ${\bar{V}}_{m}(t)$ and $\alpha_{m}$ are the target volume, which we take to be fixed in time for each *m* and the energy intensity of each cell, respectively. Note that *V*_*m*_(*t*) can be defined as below:


$$V_{m}(t)=\int_{\Omega }h(\phi_{m})dx,\;(1\leq m\leq N).$$ (2)

In Eq (2), we consider the interpolation h(ϕ):


$$h(\phi )=\phi^{2}(3-2\phi )=\left\{ \begin{array}{cc} 0 & \phi =0,\\ 1 & \phi =1,\\ \text{smooth transition} & 0<\phi <1. \end{array}\right.$$ (3)

Using h(ϕ) rather than ϕ more accurately approximates the characteristic (indicator) function of the cell domain in phase field models, improving numerical stability and volume conservation, especially near interfaces, because it minimizes interface smearing errors [55,56].

The parameter $\epsilon_{m}$ controls the thickness of the diffuse interface between different phases in the phase field model [41]. Larger values of $\epsilon_{m}$ result in a wider transition region, meaning the phase field variable changes more gradually between 0 and 1. Conversely, smaller values sharpen the interface, leading to a more abrupt transition between phases. While the total volume of each phase is theoretically preserved, wider interfaces may introduce slight numerical deviations in volume, especially if not well resolved by the computational grid. Therefore, choosing $\epsilon_{m}$ involves a tradeoff between interface resolution and numerical accuracy, and computational efficiency [57].

In our model where the sizes of the different cellular components (nurse cells, the border cell cluster, and the oocyte) vary significantly, the parameter $\alpha_{m}$ plays a critical role in enforcing volume constraints for each phase field variable $\phi_{m}$. Specially, the term $\alpha_{m}{\left(V_{m}(t)-{\bar{V}}_{m}(t)\right)}^{2}$ penalizes deviations from the target volume ${\bar{V}}_{m}(t)$, ensuring that the actual volume *V*_*m*_(*t*) remains close to the desired value. A larger $\alpha_{m}$ results in stricter volume conservation by heavily penalizing volume deviations, while smaller values of $\alpha_{m}$ allow for greater flexibility in volume variation. This term is crucial for ensuring volume preservation, which is a key aspect of the system’s dynamics.

In the next step, we consider the specific conditions of the egg chamber. To model the system, we need to consider specific conditions that reflect the biological environment of the egg chamber. Firstly, each nurse cell and the oocyte are influenced within the egg chamber by mechanical interactions with neighboring cells, particularly the compressive forces exerted by surrounding nurse cells. Additionally, the nurse cells and the oocyte must occupy adjacent but spatially distinct domains, avoiding interpenetration. The energy form of repulsion *E*_1_ can be considered as following where any overlap increases the energy in the system:


$$E_{1}=\beta_{0}\sum_{m=1}^{N}\int h(\phi_{0})h(\phi_{m})dx+\beta (m,n)\sum_{m=1}^{N}\sum_{n\neq m}^{N}\int h(\phi_{n})h(\phi_{m}),$$ (4)

where $\beta_{0}$ (epithelial-cell) and β(m,n) (cell-cell) are positive constants and represent the intensities of domain territories [41,50].

Also, the egg chamber is entirely filled with nurse cells and the oocyte, all of which are completely enclosed within the epithelial layer. Therefore, we consider *E*_2_ as below


$$E_{2}=\alpha_{0}{\left[\int_{\Omega }(1-h(\phi_{0}))dx-\sum_{m=1}^{N}V_{m}(t)\right]}^{2},$$ (5)

where $\alpha_{0}$ is the energy intensity constant for cells.

Biophysically, the term *E*_2_ enforces a global volume constraint on the interior of the egg chamber. The nurse cells and the oocyte occupy the space enclosed by the follicular epithelium, forming a tightly packed, nearly incompressible tissue compartment. Because the epithelial layer and surrounding basement membrane is mechanically rigid relative to the interior cells, and because cytoplasmic flows redistribute volume rather than create new space, the total enclosed volume can remain approximately constant during the timescale of border cell migration. The energy *E*_2_ penalizes any mismatch between the fixed interior volume (the region not occupied by epithelium, represented by $1-h(\phi_{0})$) and the sum of the volumes of the nurse cells modeled in the system. In effect, *E*_2_ prevents nonphysical expansion or compression of the tissue interior and ensures that nurse cell and oocyte volume is conserved as the border cell cluster migrates.

Cell-cell adhesion is facilitated by adhesion molecules like cadherins, which are essential for maintaining cell cohesion and for transmitting mechanical signals between cells [58–60]. For instance, the adhesion between nurse cells and the oocyte is critical for the structural integrity of the egg chamber and the coordinated migration of the border cells toward the oocyte [15,60,61]. We consider the adhesion force between nurse cells and each other and nurse cells and oocyte where opposing gradients from neighboring phases reduce the total energy


$$E_{3}=\gamma_{0}\sum_{m=1}^{N}\int \nabla h(\phi_{0})\nabla h(\phi_{m})dx+\gamma (m,n)\sum_{m=1}^{N}\sum_{n\neq m}^{N}\int \nabla h(\phi_{m})\nabla h(\phi_{n})dx,$$ (6)

where $\gamma_{0},\gamma (m,n)>0$ are the intensity of cell-epithelial adhesion and cell-cell adhesion mediated by molecules like E-cadherin [3,62,63], which are critical for maintaining cluster cohesion [60].

The total energy of the egg chamber is given by


$$E=E_{0}+E_{1}+E_{2}+E_{3}.$$ (7)

We minimize the free energy function to obtain the stable configuration of the cell cluster. Minimizing the free energy of the system ensures that the border cells cluster moves in a coordinated manner toward a stable configuration.

By taking the functional derivative of Eq (7), with respect to the $\phi_{m}$, we obtain the variational force driving the system toward energy minimization.

This derivative is then used to define a gradient flow, resulting in the time evolution equation for $\phi_{m}$, where phase equilibrium corresponds to a local minimum of the total energy


$$\frac{\partial \phi_{m}}{\partial t}=-\mu \frac{\delta E}{\delta \phi_{m}},\;(1\leq m\leq N),$$ (8)

where μ>0 is the mobility of the phase field. Evaluating the energy Eq (8) becomes


$$-\frac{1}{\mu }\frac{\partial \phi_{m}}{\partial t}=\epsilon_{m}^{2}\nabla^{2}\phi_{m}+\phi_{m}(1-\phi_{m})\left[\phi_{m}-\frac{1}{2}+6F(\phi_{m},\phi_{0})\right],\;(1\leq m\leq N),$$ (9)

in which


$$F(\phi_{m},\phi_{0})=2\alpha_{m}(V_{m}(t)-{\bar{V}}_{m}(t))+\beta_{0}h(\phi_{0})+\beta (m,n)(\xi -h(\phi_{m}))\;+2\alpha_{0}\left[\int (1-h(\phi_{0}))-\sum_{m=1}^{N}V_{m})\right]+\gamma_{0}\nabla^{2}h(\phi_{0})+\gamma (m,n)\nabla^{2}(\xi -h(\phi_{m})).$$ (10)

where $\xi =\sum_{m=1}^{N}h(\phi_{m})$.

### Initializing the equilibrium state of the egg chamber

**Fig 3:**
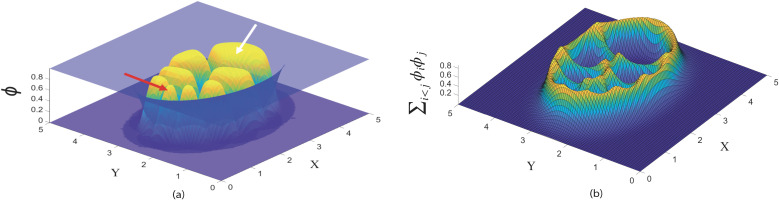
Initial state of the model before border cell cluster migration in the egg chamber. **(a)** The anterior region contains the border cell cluster (red arrow), while the posterior region contains the oocyte (white arrow), which is larger than the nurse cells. The model includes 6 nurse cells that are heterogeneous in size. Border cells, nurse cells, and the oocyte are shown in yellow. The blue surface represents the epithelial layer phase field, where $\phi_{0}=1$ inside the epithelium and $\phi_{0}=0$ outside, defining the boundary of the extracellular space. **(b)** Visualization of the overlap term $\sum_{i<j}\phi_{i}\phi_{j}$, which highlights spatial regions where individual cells physically contact each other. This quantity is *not* the total energy; rather, it identifies zones of phase-field overlap that contribute to the interaction-energy component of the model. These regions correspond to interfaces where mechanical interactions— including those associated with TIM forces—are active during migration. The parameters are set as follow: Ω=[0,5]×[0,5], size of spatial grid *h* = 0.05, the time step of Δt=0.05, $\epsilon^{2}=0.001,0.001,0.0005$ for nurse cells, oocyte, and border cell cluster, respectively. Also, $\alpha_{m}=100$ for all *m*, $\beta_{0}=0.9$, $\eta_{0}=0.007$ for epithelial layer. The adhesion intensity β(1,1)=β(1,2)=β(1,3)=β(2,1)=β(3,1)=0.25, β(2,3)=β(3,2)=0.3 and β(2,2)=β(3,3)=0. The repulsion intensity γ(1,1)=0.003,γ(1,2)=γ(2,1)=0.004, γ(1,3)=γ(3,1)=0.008, γ(2,3)=γ(3,2)=0.005, and γ(2,2)=γ(3,3)=0 (See Table A in S1 Text).

We define a two-dimensional square computational domain, Ω=[0,5]×[0,5], uniformly discretized into an *M* × *M* grid, where the constant mesh size is given by $h=\frac{5}{M+1}$. Time evolution is computed using an explicit time-stepping scheme with uniform step size Δt. The parameter values $\left(\alpha_{m},\gamma (m,n),\beta (m,n)\right)$ are chosen to reflect adhesion and volume constraints that produce biologically appropriate balance. These parameters are given in figure captions. Code for simulations is implemented in MATLAB R2022a, with a mesh size of *h* = 0.05 and a time step of Δt=0.05. Time integration is performed using an explicit scheme subject to a stability constraint of the form $\Delta t\leq \frac{1}{2\mu \epsilon^{2}}h^{2}$ [64]. We choose Δt=0.05 when *h* = 0.05, satisfying this condition for the parameter values used in this study. For finer spatial discretizations, the time step would be reduced accordingly to main stability. We use the finite difference method to solve Eq (9) with Neumann boundary conditions, which are outside of the egg chamber domain. We initialize the system by starting with small circular phases and running the simulation until *t* = 2000 and the system reaches an equilibrium where all cells have achieved their corresponding target volumes (Fig 3).

We model a 2D cross-section of the egg chamber. In this slice, six “effective” nurse cells represent the local neighbors that directly contact and confine the border cell cluster along its path. Nurse cells radii are heterogeneous to reflect packing; qualitative migration is robust to moderate changes in nurse cell number (5–8 neighbors) and size.

## Chemoattractant concentration induces migration

To investigate the impact of the spatial distribution of chemoattractants within the *Drosophila* egg chamber, we utilize a simple piecewise hyperbolic trigonometric function that captures secretion, diffusion, and degradation of chemoattractant molecules. These molecules are secreted from the anterior surface of the oocyte and form gradients that guide the directed migration of the border cell cluster through the surrounding nurse cell complex [21,23–26,37].

**Fig 4:**
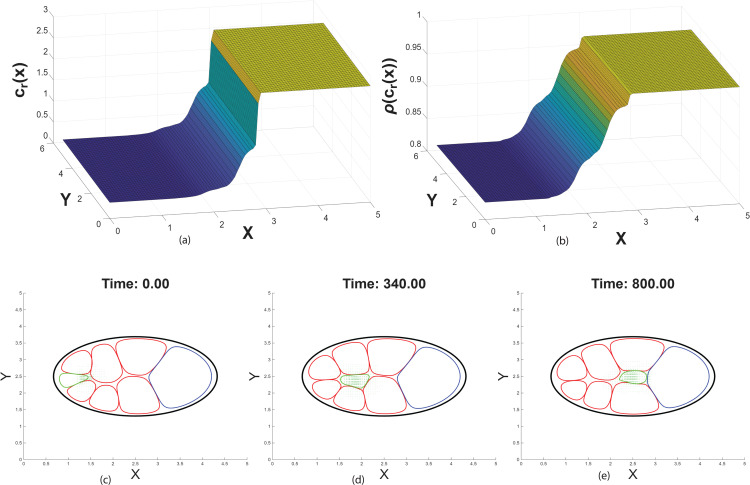
Impact of traditional chemical force term (*F*_chem_). **(a)** 2D steady-state distribution of chemoattractant concentration *c*(*x*,*y*) in the egg chamber domain. **(b)** Receptor activation in response to the chemoattractant concentration increases along the anterior-posterior axis and saturates near the posterior at the oocyte. (See S1 Fig for chemoattractant distribution without impact of radial variation)(c)-(e) Time evolution of border cell cluster migration, simulated using the phase field model in Eq (9) with **F**_chem_, Eq (12). The border cell cluster (green) initiates at the anterior end and migrates toward the posterior oocyte in response to a chemoattractant gradient. The green arrows in the cluster indicate ($\phi_{c}\nabla c$) in Eq (12). Cell boundaries are represented by phase field contours (red for nurse cells, blue oocyte, and black epithelial layer) at the ϕ=0.5 level. The parameters are set as follows: $\mu_{c}=0.045$, Γ=0.01, *s* = 1, and ℓ=0.05. The parameters used are consistent with those in Fig 3. (See S2 Fig for migration without impact of radial variation).

In George et al. [27] we show that small gaps between nurse cells can generate a heterogeneous distribution of chemoattractant along the anterior-posterior axis. There we used radial symmetry with varying radii assumption to generate a 1D model capturing the 3D volume variation. Here we assume the mediolateral space between nurse cells as 2*r*(*x*) (ultimately representing cross-sectional area used in solving Eq.(A) in S1 Text) and construct a 1D distribution with radial variation, *c*_*r*_(*x*) which we trivially extend to *c*_*r*_(*x*, *y*) for a way to capture a representation of gap-generated chemoattractant variation (see Fig 4a).

At steady state, the chemoattractant forms a spatial gradient with high concentration near the oocyte surface and lower levels toward the anterior side of the egg chamber [16,21,37]. The border cell cluster senses this gradient through highly-regulated receptor-mediated signaling and polarizes in response to local concentration differences [23,24,26,28–31,65–67]. Although the chemoattractant gradient is temporally constant, spatial asymmetry in its distribution drives directed migration: cells at the leading edge of the cluster experience higher chemoattractant levels than those at the rear, resulting in coordinated forward motion [15,16,21,37].

Receptor-mediated signaling enables the border cell cluster to interpret extracellular chemoattractant concentrations and initiate directed migration. We assume receptor activation depends nonlinearly on the local chemoattractant concentration *c*, capturing cooperative effects and saturation behavior. We use receptor dynamics ρ(c) in functional form of [27]:


$$\rho (c)=\frac{sc^{3}}{\left(c^{2}+\Gamma )(c+\ell \right)},$$ (11)

where *s* is the maximal activation level, and Γ and ℓ control the sensitivity of the response (Fig 4b).

The receptor activation function Eq (11) is inherited from our previous receptor–chemoattractant model for Drosophila border cells [27] and can be understood as a coarse-grained, steady-state solution of a three-compartment receptor system. In that model, receptors cycle between localized at the cell surface and active ($\rho_{a}$), non-active at the surface ($\rho_{n}$), and non-active and internalized ($\rho_{i}$) states with activation, recycling, and degradation rates that depend on the local chemoattractant concentration *c* (see Eqs (S1.5)–(S1.7) and S3 Fig in [27]). Under a quasi–steady-state approximation for receptor trafficking and assuming (i) cooperative activation $w(c)=\hat{w}c^{2}$ and (ii) chemoattractant-dependent recycling $\beta (c)=\hat{\beta }c$, the steady active receptor level takes the rational form


$$\rho_{a}(c)=\frac{\alpha \hat{w}\hat{\beta }c^{3}}{\Gamma (\hat{w}c^{2}+\Gamma )(\hat{\beta }c+\hat{\Gamma })}=\frac{s\;c^{3}}{\left(c^{2}+\Gamma )(c+\ell \right)},$$

after rescaling and grouping parameters as s=α/Γ, $\Gamma =\Gamma /\hat{w}$, and $\ell =\hat{\Gamma }/\hat{\beta }$ (cf. Eq (S1.9) in [27]). Thus, Eq. (11) represents the steady-state fraction of active receptors in a receptor trafficking/activation model with cooperative binding and ligand-dependent recycling.

Biophysically, ρ(c) behaves like a Hill-type activation function with effective cooperativity of order three: the numerator *c*^3^ encodes ultrasensitive growth with increasing chemoattractant, while the denominators $\left(c^{2}+\Gamma \right)$ and (c+ℓ) produce smooth saturation and set characteristic concentration scales. The parameter *s* > 0 sets the maximal level (gain) of effective receptor activation, while Γ>0 and ℓ>0 determine the approximate half-activation concentration and the width of the transition region, playing roles analogous to effective dissociation constants in standard dose–response curves. As in [27], we choose (s,Γ,ℓ) so that ρ(c) is negligible at anterior basal concentrations, steeply increasing along the migration path, and saturated near the posterior of the egg chamber; our qualitative conclusions are robust to variations of these parameters within this regime.

### Traditional chemoattractant force

Previous work has the chemotactic contribution to a phase-field system given by the term


$${𝐅}_{\text{chem}}=-\mu_{c}\nabla \cdot (\phi_{c}\nabla c),$$ (12)

where $\mu_{c}$ is the chemoattractant sensitivity for the phase field variable of border cell cluster $\phi_{c}$, and *c* denotes the concentration of the chemoattractant [41]. This force shows how the cluster phase variable $\phi_{c}$ responds to spatial gradients in the chemoattractant concentration. The variation in the vectorfield (divergence) of the phase field, $\phi_{c}$, and the gradient of the chemoattractant, ∇c, drives the movement of the cluster toward higher concentrations of the chemoattractant. The strength of this response is modulated by $\mu_{c}$, which determines how strongly the cluster is influenced by the chemoattractant.

We add the chemical force (**F**_chem_) to the right-hand side of the phase-field evolution Eq (9). Fig 4c–4e illustrates the migration of the border cell cluster through the extracellular spaces between nurse cells. First, the chemoattractant reaches a steady-state distribution within the extracellular domain. At this stage, the border cell cluster senses the concentration gradient and initiates directed migration. In addition to the chemotactic force induced by the oocyte-secreted chemoattractant, the cluster relies on adhesion and repulsion forces arising from interactions with the nurse cells and the oocyte. As the cluster migrates, it experiences spatially varying mechanical and chemical cues, which influence its speed. Nearer to the oocyte, receptor saturation occurs due to the elevated chemoattractant concentration, resulting in a reduction in migration speed and eventual attachment of the cluster to the oocyte.

### Tangential Interface Migratory (TIM) Force

Experimental studies, including live imaging and adhesion molecule manipulation, indicate that border cell migration within the *Drosophila* egg chamber involves more than chemotactic guidance or direct propulsion through nurse cells. The cluster frequently displays tangential behaviors—such as spreading, sliding, and crawling—along nurse cell surfaces [22,28,29,32,33,68]. These behaviors are accompanied by dynamic, actin-rich protrusions and transient cell-cell contacts, pointing to a critical role for mechanical interactions at the cluster–nurse cell interface. Prasad and Montell [28] observed lateral and rearward protrusions and dynamic positional rearrangements, supporting the idea of contact-mediated motion. Aranjuez et al. [22] and Mishra et al. [33] further showed that border cells generate traction via localized actomyosin contractility and Myo-II enrichment at the interface with nurse cells, highlighting the role of spatially organized mechanical forces in collective migration. These observations collectively support the existence of shear-like, tangential traction forces not captured by normal adhesion or repulsion alone. To represent this mechanism mathematically, we introduce a novel force term—the *Tangential Interface Migration (TIM)* force—which captures the directionally biased, contact-mediated propulsion generated as border cells use surrounding nurse cells as mechanical substrates for forward migration [22,23,60,61].

**Fig 5:**
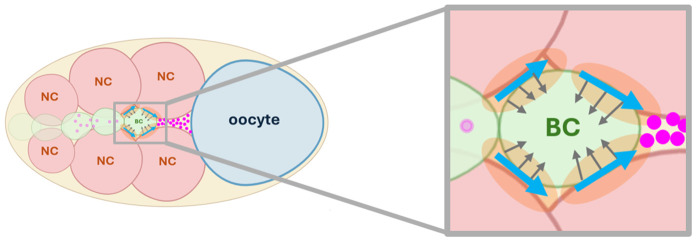
Schematic representation of Tangential Interface Migration (TIM) force. The border cell cluster (BC, green) is surrounded by nurse cells (NC, pink), the regions of contact between the border cell cluster and nurse cells are shown in orange representing the overlap of phases (border cell cluster phase variable $\phi_{c}$ and nurse cell phase variables $\phi_{j}$). The small gray arrows show the gradient of cluster phase $\nabla \phi_{c}$ and large blue arrows show the tangential vectors corresponding to the vectors orthogonal to the gradient ($\nabla \phi_{c}{)}^{⟂}$. Chemoattractant molecules (magenta dots) indicate that the presumed concentration at the front of cluster (posterior side) is more than at the back of the cluster (anterior side), which amplifies the movement of cluster from the anterior to the posterior.

The TIM force represents contact-mediated, directionally biased traction generated at the cluster-nurse cell interface. Unlike traditional force terms that model repulsion or isotropic adhesion, the TIM force captures the ability of the border cell cluster to “crawl” or “climb” along adjacent nurse cells (Fig 5). We define this force function as below:


$${𝐅}_{\text{TIM}}=-{\bar{\mu }}_{c}\nabla \cdot \left(\rho (c)\;\phi_{c}\phi_{j}\;\mathrm{sgn}(\nabla c\cdot \nabla \phi_{c}^{⟂})\nabla \phi_{c}^{⟂}\right),$$ (13)

where $\phi_{c}$ and $\phi_{j}$ are the phase field variables representing the cluster and a neighboring nurse cell, respectively. The constant parameter ${\bar{\mu }}_{c}$ represents the strength of TIM force. The product $\phi_{c}\phi_{j}$ identifies as an overlap, the interfacial region where the border cell cluster is in contact with a neighboring nurse cell. The vector $\nabla \phi_{c}^{⟂}$ denotes the tangential direction along the cluster boundary. The sign function $\mathrm{sign}(\nabla c\cdot \nabla \phi_{c}^{⟂})$ determines the orientation of the tangential force based on the local alignment between the chemoattractant gradient and the cluster boundary. Specifically, it selects the direction along the tangential interface that is aligned with the increase in chemoattractant concentration. This allows the TIM force to promote movement in the direction of higher chemoattractant levels while preserving geometric consistency along the boundary.

Note that we can also write the TIM force instead of the divergence of a vector field as the inner product of the two vectors fields:


$${𝐅}_{\text{TIM}}=-{\bar{\mu }}_{c}\nabla \left(\rho (c)\;\phi_{c}\phi_{j}\right)\cdot \left(\mathrm{sgn}(\nabla c\nabla \phi_{c}^{⟂})\nabla \phi_{c}^{⟂}\right),$$ (14)

which shows the gradient alignment of chemoattractactant through the receptor activation function, ρ, with the tangential vector field is key to migration progression. The term $\nabla \left(\rho (c)\;\phi_{c}\phi_{j}\right)$ captures the spatial variation in receptor-ligand interactions along the contact interface, where ρ(c) represents a receptor-mediated response to the chemoattractant concentration *c*. Eq (14) captures the alignment between the gradient of receptor-regulated interfacial contact and the local tangential direction of the cluster boundary. The direction of the TIM force is mechanically constrained by cell geometry, while its magnitude is modulated by spatial variations in chemoattractant concentration through receptor-ligand interactions.

We considered the case of a uniform chemoattractant field (∇c=0), which removes directional chemical guidance. Under symmetric initial conditions and uniform mechanical parameters, the border cell cluster exhibited only small, non-persistent shape fluctuations and no net displacement of its centroid. Migration did not occur unless a spatial gradient in *c* was present, indicating that in the model, as in experiments, directional guidance requires asymmetry in external signaling.

**Fig 6:**
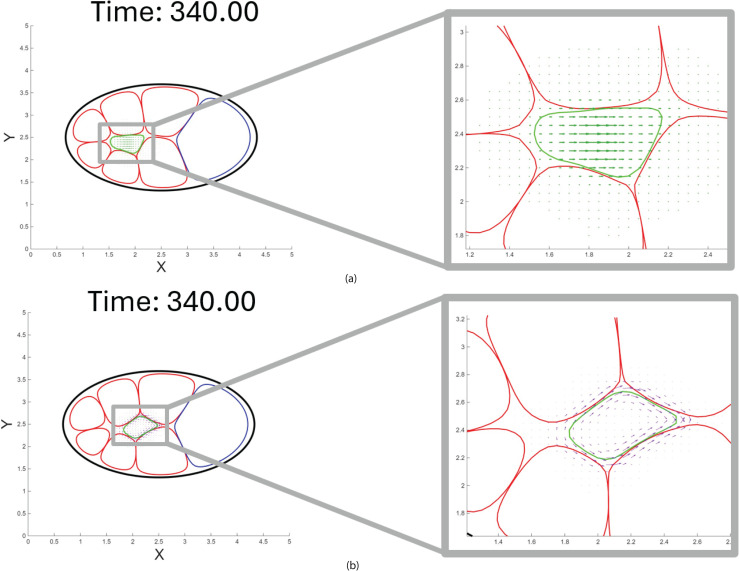
Comparison of *F*_chem_ and *F*_TIM_ vector fields. Vector fields prior to taking the divergences in each of the chemotactic forms. **(a)** Visualization of the chemotactic force vector field (*F*_chem_), which directs the cluster toward regions of higher chemoattractant concentration. The magnitude and orientation of the force vectors vary in response to spatial changes in the chemoattractant. **(b)** Visualization of the tangential interface migration force (*F*_TIM_), which is generated by interfacial interactions between the cluster and adjacent nurse cells. The enlarged panel shows tangentially oriented vectors along the cluster boundary, capturing contact-mediated propulsion. In both panels migration is shown at the same time *t* = 340; nurse cell boundaries are denoted by red; black outlines the epithelial layer of the egg chamber; the blue contour marks the oocyte; and the green region represents the border cell cluster.

The chemical force (Eq (12)) on the right-hand side of the phase-field evolution Eq (9) is replaced by the TIM force (Eq (13)). Fig 6a shows the spatial localization and orientation of the chemical force during border cell migration. The green arrows in the right panel represent the magnitude and direction of the chemical force, which is spatially localized due to the chemoattractant concentration gradient. In Fig 6b, the left panel displays the border cell cluster (green interface) in contact with nurse cells (red interfaces), with purple arrows indicating the tangential interface migration vectors localized at the overlapping regions and point from the anterior to the posterior, consistent with the chemoattractant gradient and the cluster’s net migration direction. The right panel magnifies this interaction zone, showing that the TIM force is spatially restricted to areas where the border cell cluster overlaps with nurse cells and aligns tangentially to the cell boundary, reinforcing the notion of contact-guided movement.

**Fig 7:**
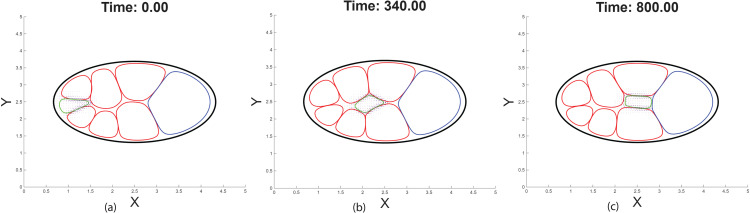
Impact of Tangential Interface Migration force (*F*_TIM_). Snapshots of the cell configuration and TIM force vector field at three time points (a) *t* = 0: The cluster is initially positioned near the anterior of the egg chamber and begins contact-mediated migration along adjacent nurse cell interfaces. Forward movement is initiated as the leading edge of the cluster experiences higher chemoattractant concentration, as indicated by the purple arrow at the front. (b) *t* = 340: The cluster is midway through its migration path, exhibiting tangential traction along adjacent nurse cell boundaries. Vectors indicate directed force aligned with the interfaces. (c) *t* = 800: The cluster approaches the oocyte boundary. At this stage, the front of the cluster senses reduced chemoattractant signaling due to receptor saturation, leading to a gradual decrease in migration speed and eventual attachment to the oocyte.

**Fig 8:**
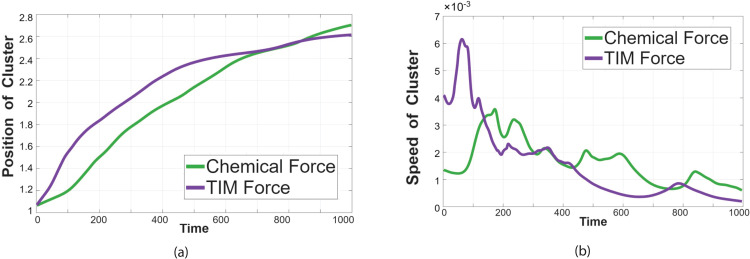
Comparison of border cell cluster migration under *F*_chem_ and *F*_TIM_. **(a)** Temporal evolution of the center of cluster’s position along the anterior-posterior axis. **(b)** Speed of the border cell cluster during migration along the anterior–posterior (A–P) axis.

To further analyze the effect of the TIM force (**F**_TIM_) on migration behavior, we simulate the time evolution of the cluster and track its movement under the influence of TIM. As shown in Fig 7a–7c, the panels present the migration of the cluster progressing through the extracellular space by engaging with and pushing off of adjacent nurse cells. When we replace the traditional chemotactic force *F*_chem_ with the TIM force, the cluster still migrates, demonstrating that TIM provides a chemoattractant-responsive, contact-mediated mechanism that does not rely on the gradient magnitude used in classical chemotaxis. In Fig 8a, the cluster under TIM force (purple) migrates more rapidly in the early phase and reaches the oocyte boundary earlier compared to the cluster guided by chemotactic force (green), which progresses more gradually. Fig 8b shows cluster speed over time. The TIM-driven cluster exhibits an early peak in speed, followed by a gradual decline as it approaches the oocyte, likely due to receptor saturation and reduced interfacial traction. In contrast, the chemotactic force leads to more variable speed with multiple transient peaks, reflecting sensitivity to changes in the chemoattractant gradient shaped by the extracellular geometry (See Fig C, D, and E in S1 Text and S5–S8 Movies for effects from radial variation of extracellular space on migration characteristics.). These trends follow from the force definitions. TIM is the divergence of a tangential traction density confined to the outer cluster–nurse–cell interface; its resultant scales with the length and alignment of the contacting arc and the chemo-dependent gain ρ(c), yielding an early speed peak that declines as the A–P–aligned contact shortens/curves and ρ(c) saturates near the oocyte. This is consistent with observations *in vivo* that the border cell cluster migrates faster initially and then slows later, on average (See Fig F in S1 Text and [27,29,52]). By contrast, $F_{chem}=-\mu_{c}\nabla \;\cdot (\phi_{c}\nabla c)$ drives motion up-gradient and does not depend on contact length; as the extracellular geometry reshapes the gradient, the magnitude/direction vary, producing the variable speed with transient peaks and dips.

Unlike the chemotactic force *F*_chem_, whose magnitude scales directly with the local gradient |∇c|, the TIM force is designed to represent a distinct biophysical mechanism: contact-mediated traction along a border cell–nurse cell interface that is engaged once chemoattractant receptors locally are sufficiently stimulated. In this setting, the direction of motion is determined by the sign of the projected gradient $\mathrm{sgn}(\nabla c\cdot \nabla \phi_{c}^{⟂})$, while the magnitude of the traction is governed primarily by the receptor activation level ρ(c) and the available interface $\phi_{c}\phi_{j}$. Because ρ(c) is a saturating, Hill-type function of the local concentration rather than its gradient, TIM can generate approximately order-one tangential traction in regions where |∇c| is shallow but *c* is sufficiently high for receptor activation to plateau. Thus, TIM does not assume that force vanishes in flat or concave regions of *c*; instead, it models the experimentally observed behavior in which posteriorly activated interfaces continue to produce traction even when the gradient becomes weak. This explains why TIM can maintain migration in profiles where *F*_chem_ naturally slows or stalls.

The TIM force is designed to model a class of contact-mediated guidance mechanisms that operate specifically along the border cell–nurse cell interface. Its core factor, $\rho (c)\phi_{c}\phi_{j}$, represents the effective density of active receptor–ligand pairs within the region where the border-cell phase field $\phi_{c}$ overlaps with that of an adjacent nurse cell $\phi_{j}$. In biological terms, this quantity corresponds to the local abundance of engaged adhesion molecules such as DE-cadherin, which are known to cluster within micrometer-scale interfacial patches, transmit physical forces, and link to actin-assembly machinery activated by signaling pathways on sub-minute timescales. The product $\phi_{c}\phi_{j}$ restricts the force to the geometric interface, while ρ(c) introduces dependence on chemoattractant-mediated receptor activation, consistent with observations that signaling pathways (e.g., PVR and EGFR) modulate cadherin tension and actomyosin enrichment at specific boundary regions [23,29,37,60,69].

The direction of the TIM force is encoded by the term $\mathrm{sgn}(\nabla c\nabla \phi_{c}^{⟂})\nabla \phi_{c}^{⟂}$, which selects a tangential orientation along the interface rather than a normal pushing force. This captures the experimentally observed phenomenon [21,22,28,29,33,60,70] that border cells preferentially extend protrusions and generate traction along chemically biased stretches of their perimeter, rather than rather than in lateral directions. Thus TIM represents neither whole cell protrusive crawling nor a symmetric friction effect. Instead, it models a localized shear-like traction that arises when receptor activation is spatially asymmetric along an interface, leading to directed tangential motion of the cluster. Similar contact-induced steering has been reported in border cell migration, where receptor signaling modulates cortical contractility and biases adhesion and actin organization at cell-cell boundaries [22,31,33,60,70].

This formulation, therefore, provides a mechanochemical bridge linking localized receptor activation, interfacial cytoskeletal remodeling, and the emergent tangential traction that contributes to coherent cluster migration.

## TIM force reproduces Gurken‑driven dorsal guidance

When the cluster is close to the anterior surface of the oocyte it executes a ∼ 10−15μm “dorsal turn” toward the germinal vesicle. Genetic studies show that this second phase of migration is driven by a short‑range epidermal growth‑factor–receptor (EGFR) cue that originates from the oocyte nucleus [24,26]. Gurken (Grk), a TGF‑α-like ligand, accumulates as a crescent near the dorsal‑anterior oocyte nucleus [71] and activates EGFR in the border‑cell cluster [31]. Eliminating EGFR activity in border cells or mutating *grk* abolishes the dorsal movement, but mutating other guidance factors does not [24,26,66]. Because Grk protein diffuses only a short distance, its effect is confined to border cells in close proximity to the oocyte surface. Earlier anterior to posterior migration, by contrast, relies on the long‑range PDGF/VEGF ligand PVF1 signaling through PVR, and to some extent on other EGFR ligands besides Grk, but these latter proteins are believed to be expressed in spatiotemporal patterns inconsistent with dorsal cues [21,24,26,37].

**Fig 9:**
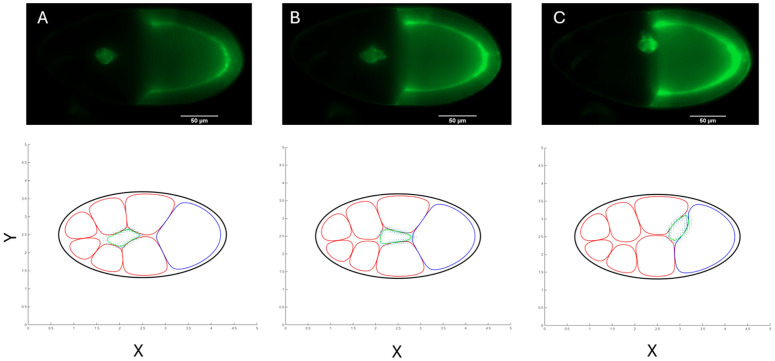
The short‑range Gurken cue drives the dorsal turn of the border‑cell cluster: comparison of live imaging and phase‑field simulation. Top row: Time-lapse images of the egg chamber expressing a membrane localized GFP marker (green), which shows the border cell cluster and outlines the oocyte. The border‑cell cluster (bright spot) migrates toward the right **(A)**, and as it nears the surface of the oocyte, reorients dorsally toward the germinal vesicle (B) and continues along the anterior side of the oocyte towards the dorsal side of the egg chamber (top) **(C)**. Bottom row: Corresponding snapshots from the phase‑field model at the indicated simulation times (*T* = 258, 426, 1311). The arrows depict the TIM force, which is proportional to the local chemoattractant gradient *c*(*x*,*y*). The shallow dorsal component of *c* becomes appreciable once the cluster contacts the oocyte, redirecting TIM stresses upward and reproducing the experimentally-observed dorsal migration without additional parameter tuning.

To produce this two-step chemotactic landscape, we prescribe a steady concentration field $c(x,y)=\cosh(1.5x)+10^{-4}\cosh(y)$, with *x* and *y* denoting the anterior-posterior (AP) and medio-lateral axes, respectively. The AP component cosh(1.5x) yields a steep PVF1-like gradient that pulls the cluster through the nurse cell matrix. The dorsal component $10^{-4}\cosh(y)$ introduces a shallow dorsal bias, though present throughout the chamber, that becomes appreciable only after the cluster reaches the oocyte, mimicking the short-range Grk signal. Within our framework the TIM force converts local chemoattractant gradients into interfacial traction. Consequently, as soon as the cluster contacts the oocyte, the now-dominant dorsal gradient redirects the tangential stresses dorsally, recreating the experimentally-observed dorsal excursion shown in Fig 9 (See also S1–S4 Movies). No additional parameter tuning, beyond that for the primary AP axis cues representing PVF1, is required, supporting the view that a localized Gurken cue, together with interfacial mechanics, drives the dorsal turn.

We note that although the analytic field $c(x,y)=\cosh(1.5x)+10^{-4}\cosh(y)$ attains larger absolute values than the physiologically motivated profile in Fig 4, this does not increase the magnitude of the TIM force, because ρ(c) is a saturating activation function. Once *c* exceeds the activation threshold, ρ(c) (and hence the magnitude of *F*_TIM_) remains essentially constant. We use this analytic form only to create a controlled shallow-gradient region in which the qualitative differences between chemotaxis and TIM can be clearly illustrated.

## TIM force enables migration under a shallower chemoattractant gradient

*In vivo* chemoattractant gradients guiding collective cell migration are often shallow, heterogeneous, and spatially distorted by tissue geometry. Diffusion, degradation, and variations in extracellular space can substantially reduce the local magnitude of ∇c, even while preserving a consistent anterior–posterior bias in ligand concentration. Experimental studies of border cell migration and other collective systems indicate that directed movement can persist under such weak or noisy chemical cues, suggesting that additional guidance mechanisms beyond classical gradient sensing must contribute to robust migration [3,13,21,22,27,28,37,60].

To directly test the consequences of weaker chemoattractant signaling, we compared border cell cluster migration driven by the traditional chemotactic force *F*_chem_ and by the tangential interface migration (TIM) force *F*_TIM_ under chemoattractant profiles with reduced gradient magnitude. In these simulations, the chemoattractant concentration remains asymmetric across the cluster but exhibits a shallow spatial slope, corresponding to conditions where directional bias is preserved but the local gradient magnitude is too small to generate strong chemotactic propulsion.

**Fig 10:**
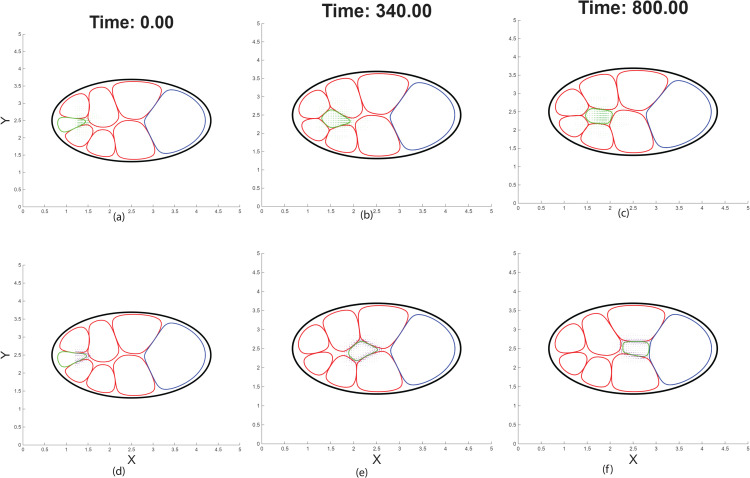
Comparison of chemotactic and tangential interface migration (TIM) forces under weak chemoattractant gradients. **(a–c)** Time evolution of border cell cluster migration driven by the classical chemotactic force *F*_chem_ under a shallow chemoattractant gradient, shown at *t* = 0, *t* = 340, and *t* = 800. As the cluster enters regions where the gradient magnitude |∇c| is reduced, the chemotactic force weakens and the cluster stalls midway through the egg chamber, failing to reach the oocyte. **(d–f)** Corresponding snapshots for migration driven by the tangential interface migration (TIM) force *F*_TIM_ under the same shallow-gradient conditions and at the same time points. Despite the shallow chemoattractant profile, the cluster maintains directed motion and successfully completes migration to the oocyte. TIM-generated traction is localized to border cell–nurse cell interfaces and depends on receptor activation and interfacial contact rather than on the magnitude of the gradient, allowing migration to proceed when classical chemotaxis fails.

Under these conditions, migration driven by the chemotactic force $F_{chem}=-\mu_{c}\nabla \cdot (\phi_{c}\nabla c)$ fails to complete. As the cluster enters regions where the gradient flattens, the chemotactic force decreases in magnitude and eventually becomes insufficient to overcome mechanical constraints imposed by neighboring nurse cells (Fig 10b). Consequently, the border cell cluster slows and stalls midway through the egg chamber, despite the continued presence of a posterior–anterior chemoattractant bias (Fig 10c). This behavior reflects a fundamental limitation of gradient-based chemotaxis: when propulsion strength scales directly with |∇c|, shallow or concave profiles can suppress forward motion even when directional information is still present.

**Fig 11:**
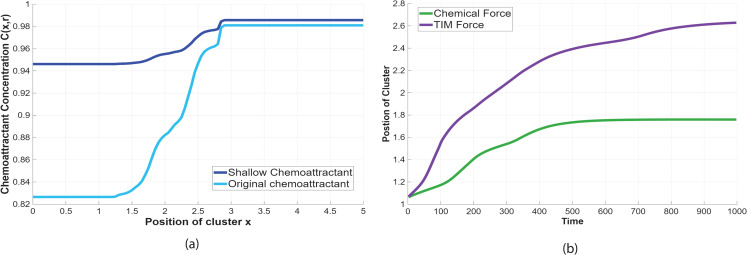
Effects of reduced chemoattractant secretion on gradient profile and migration outcome. **(a)** Comparison of the original chemoattractant concentration profile and a shallower profile generated by reduced secretion from the oocyte. The shallow profile preserves anterior–posterior asymmetry but exhibits a substantially reduced spatial gradient. **(b)** Cluster position over time under the shallow gradient. Migration driven by the chemotactic force stalls prematurely (green), whereas migration driven by the TIM force successfully completes migration to the oocyte (purple).

The chemoattractant profiles used in these simulations are shown explicitly in Fig 11a. Reducing secretion from the oocyte produces a markedly shallower chemoattractant profile compared to the original condition, lowering the local gradient magnitude experienced by the migrating cluster while preserving an overall anterior–posterior concentration bias. Although directional information is retained, the reduced slope substantially weakens gradient-based force generation.

The consequences of this shallower gradient for migration are quantified in Fig 11b, which compares the cluster’s position over time under classic chemotactic and TIM-driven chemotatic migration. Under the weakened gradient, migration driven by the classic chemotactic force stalls prematurely (green curve), with the cluster failing to traverse the egg chamber. In contrast, when the same shallow chemoattractant profile is coupled to the TIM force, the cluster continues to advance and successfully completes migration to the oocyte (purple curve). This divergence highlights that while classical chemotaxis depends critically on gradient steepness, TIM can sustain directed motion under weaker chemoattractant conditions by converting modest chemical bias into persistent tangential traction along cluster–nurse cell interfaces. Prasad and Montell [28] observed lateral and rearward protrusions and dynamic positional rearrangements, supporting the idea of contact-mediated motion. Aranjuez et al. [22] and Mishra et al. [33] further showed that border cells generate traction via localized actomyosin contractility and Myosin-II enrichment at the interface with nurse cells, highlighting the role of spatially organized mechanical forces in collective migration. These observations collectively support the existence of shear-like, tangential traction forces not captured by normal adhesion or repulsion alone. To represent this mechanism mathematically, we introduce a novel force term—the *Tangential Interface Migration (TIM)* force—which captures the directionally biased, contact-mediated propulsion generated as border cells use surrounding nurse cells as mechanical substrates for forward migration [22,23,60,61].

In contrast, when migration is driven by the tangential interface migration force, the border cell cluster successfully completes migration under the same shallow -gradient conditions. Because the TIM force depends on receptor activation ρ(c) and on the availability of cluster–nurse cell interfaces rather than on the magnitude of the gradient itself, it generates sustained tangential traction once chemoattractant levels exceed the activation threshold. The direction of motion is determined locally by the sign of the projected gradient along the interface, while the magnitude of traction remains approximately constant due to receptor saturation. As a result, the cluster continues to advance, ultimately reaching and attaching to the oocyte (Fig 10d–10f).

These results demonstrate that TIM provides a robust, contact-mediated mechanism for collective migration in environments where classical chemotaxis is ineffective. In shallow or geometrically distorted chemoattractant fields, TIM converts weak chemical bias into sustained mechanical traction along cell–cell interfaces, enabling directed movement without requiring steep gradients. This behavior is consistent with experimental observations of border cell sliding, crawling, and traction generation along nurse cell surfaces [21,22,28–30,33,68,70] and highlights the importance of interfacial mechanics in collective guidance under physiologically realistic signaling conditions.

## Discussion

The coordinated migration of the border cell cluster in the *Drosophila* egg chamber offers a powerful model for dissecting the biophysical principles underlying collective cell motility. While chemoattractant signaling is known to play a dominant role in guiding migration, recent experimental observations suggest that mechanical interactions with the surrounding tissue also influence both the directionality and dynamics of the cluster’s movement. In this study, we developed a multi-cellular phase-field model that captures key aspects of this process, including extracellular chemoattractant dynamics, receptor-mediated signaling, and contact-mediated mechanical forces.

One of the central contributions of our work is the introduction of the Tangential Interface Migration (TIM) force, which models the ability of the border cell cluster to generate propulsion by engaging tangentially with the surfaces of adjacent nurse cells. This force is motivated by live-imaging studies showing lateral protrusions, cell-on-cell sliding, and traction generation at the cluster–nurse cell interface [22,28,29,32,68]. Our simulations demonstrate that the TIM force alone is sufficient to drive forward migration through confined spaces. This supports a view in which chemical and mechanical cues act in parallel or even partially redundantly to ensure robust guidance under variable tissue conditions.

The chemoattractant distribution in our model is shaped by the geometry of the extracellular space, which acts as the domain through which the signal diffuses and degrades. Variations in the width and structure of this space—particularly at cell-cell junctions and intersections—can significantly alter the shape and steepness of the resulting gradient. As shown in George et al. [27], local expansions or intersections in the extracellular domain lead to a reduction in gradient steepness due to increased spatial dilution and slower accumulation of the signal. This effect diminishes the directional cue available to migrating cells, resulting in a measurable decrease in migration speed within these regions. Our current model reinforces the idea that not only the source and decay rates, but also the geometry of the extracellular environment, play a critical role in shaping effective chemotactic guidance.

Importantly, our results suggest that the border cell cluster’s ability to interpret shallow or spatially irregular gradients depends on its capacity to generate traction at interfaces. In shallow-gradient regions, such as during the dorsal turn, the TIM force maintains directional progression and cluster cohesion even when the traditional chemotactic force becomes weak. This aligns with prior work showing that actomyosin contractility and E-cadherin-mediated adhesion at the cluster periphery are necessary for coordinated migration [22,33,60,70]. The TIM force provides a biophysically based framework to account for these contact-based behaviors in a continuum model, offering an alternative to purely chemotactic guidance under certain conditions.

Our results demonstrate that the Tangential Interface Migration (TIM) force offers several advantages over classical chemotactic force such as *F*_chem_.

First, TIM requires direct contact between the border cell cluster and the nurse cells to initiate movement, migration does not occur without sufficient overlap at the interface, reflecting the physical necessity of a substrate for generating tangential traction. Second, TIM drives motion tangential to the border cell–nurse cell interface, capturing mechanical behaviors such as sliding and wrapping, as observed in live imaging studies but not modeled by classical chemotactic approaches. Third, TIM supports persistent, cohesive migration even in regions where the chemoattractant gradient is weak or flattening, conditions under which the chemotactic force *F*_chem_ can lead to stalled or erratic movement. This behavior follows from the analysis in Fig 10: chemotactic forcing vanishes when the gradient flattens, but TIM aggregates receptor activation along the cell-cell interface and responds to the direction rather than the strength of the gradient. As long as the profile still varies consistently across the interface, TIM continues to generate directed traction even when *F*_chem_ fails. For example, if the chemoattractant distribution has negative concavity, *F*_chem_ may stall or even go negative though the chemoattractant gradient may still be positive. This cannot happen with *F*_TIM_. Together, these characteristics make TIM a robust and biophysically grounded mechanism for guiding collective migration through the densely packed and heterogeneous environment of the nurse cell complex.

In this work we model the entire border-cell cluster using a single phase field $\phi_{c}$, which treats the 6–8 cells as a mechanically cohesive unit. This formulation enables us to focus on the collective interfacial mechanics that drive cluster-scale motion, but it necessarily omits intra-cluster rearrangements, leader–follower transitions, and heterogeneity in receptor expression or cell cortex contractility. A fully multi-cell representation where each border cell is assigned its own phase field would allow internal reorganization, spontaneous leader emergence driven by localized signaling, and competition among cells for exposure to the chemoattractant. Such extensions could modify the detailed pattern of protrusion dynamics or the spatial distribution of traction within the cluster. However, the core results presented here, including the comparison between chemotactic and tangential interfacial guidance and the robustness of the TIM mechanism in shallow or concave chemoattractant profiles, rely primarily on the geometry and mechanics of the cluster nurse cell interface rather than on internal cluster rearrangements. We therefore expect the qualitative advantage of the TIM version of chemotaxis, and the overall directionality of migration, to persist in a multi-cell formulation, even though the fine-scale organization of forces within the cluster would necessarily become richer. Future work incorporating explicit cell–cell boundaries within the cluster will be valuable for examining how leader selection and heterogeneous receptor distributions interact with the TIM mechanism.

We also prescribed a steady chemoattractant profile *c*(*x*,*y*) rather than solving a fully time-dependent diffusion–reaction equation. Biologically, this reflects a quasi–steady-state approximation in which oocyte-secreted ligands such as PVF1 have already equilibrated within the extracellular space on a timescale faster than that of border cell migration, so that the cluster experiences an effectively stationary posterior–anterior gradient shaped by tissue geometry. This assumption allows us to focus on the mechanical comparison between the traditional chemotactic force *F*_chem_ and the tangential interface migration (TIM) force *F*_TIM_ under a fixed, heterogeneous guidance landscape. A limitation of this approach is that we do not capture transient buildup or depletion of ligand, nor feedback of cluster motion on the chemoattractant field.

Overall, this work advances our understanding of how border cell clusters integrate chemical and mechanical information during migration. The TIM force offers a biologically grounded mechanism for contact-mediated movement that complements existing models of chemotaxis. More broadly, our results illustrate the value of phase-field methods for modeling migration in structured tissues, where cell-cell interfaces and spatial constraints are critical determinants of migratory behavior.

Our formulation of the TIM force places this work within a broader landscape of collective migration models that combine chemical and mechanical cues. While classical chemotaxis models assume that propulsion strength scales with the chemoattractant gradient, interface-based mechanisms such as contact inhibition of locomotion (CIL), as recently modeled in a multiphase-field framework by Monfared et al. [72], demonstrate that contact-mediated polarity cues can also promote cell movement. TIM is distinct from both approaches: it represents a chemoattractant-dependent, adhesion-regulated tangential traction generated specifically at the interfaces between migratory cells and their substrates. Thus TIM does not replace chemotaxis or CIL mechanistically. It occupies an intermediate conceptual space in which external chemical cues modulate the mechanical engagement of interfacial adhesion rather than directly setting propulsion strength.

Another modeling choice concerns autonomous propulsion. Many multiphase-field models include a cell-intrinsic propulsion term, but experimental studies of border cell migration indicate that motion arises primarily from adhesion-mediated traction within the cluster, not from single-cell lamellipodial crawling. In accordance with these observations, our model does not use a baseline propulsion force and instead focuses on how chemoattractant-regulated interfacial mechanics drive collective motion.

## Supporting information

S1 Text Supplemental Document S1_Text.pdf. (PDF)

S1 MovieLive border cell migration in a normal genotype.Border cells (green) migrate in a wild-type egg chamber towards the oocyte, as seen by live time-lapse imaging *ex vivo*. Genotype: slbo-Lifeact-GFP; matα-Gal4 / UAS-Myr-tdTomato. Only the GFP is shown. 1 hour and 20 minutes into the movie, the border cells nearly reach the oocyte boundary and then turn to move dorsally (upwards) to be close to the oocyte nucleus, which is the source of the chemoattractant signal Grk. Time stamp = hrs:min. Scale Bar = 50 μm. See Fig 9 for a representative time point from this simulation.(MP4)

S2 MovieModel border cell cluster migration in response to the chemoattractant concentration gradient (*F*_chem_).Parameters are set as follows: $\mu_{c}=0.045$, Γ=0.01, *s* = 1, and ℓ=0.05. In this simulation, the cross-sectional area between nurse cells satisfies *A*(*x*) > 0 and is incorporated into the chemoattractant concentration model. The dynamics shown here are illustrated in Fig 4c–4d of the main text.(AVI)

S3 Movie Model border cell cluster migration in response to the Tangential Interface Migration (TIM) force (*F*_TIM_). The arrows indicate tangentially oriented force vectors along the cluster boundary. A corresponding snapshot is shown in Fig 7 in the main text.
(AVI)

S4 Movie Dorsal turn of the border cell cluster. The arrow shows the tangential interface migration force, proportional to the local chemoattractant concentration. The simulation corresponds to Fig 9 in the main text.
(AVI)

S5 Movie Model border cell cluster migration in response to the chemoattractant gradient when the cross-sectional area is fixed at *A*(*x*) = 1, corresponding to a constant radius *r.* The cluster migrates in response to the chemical gradient. This simulation provides the context for the results presented in Fig B in S1 Text.
(AVI)

S6 Movie Migration of the model border cell cluster under the influence of the TIM force when the cross-sectional area is fixed (*A*(*x*) = 1). This simulation corresponds to Fig D in S1 Text.
(AVI)

S7 Movie Border cell cluster migration under chemical force when the chemoattractant concentration gradient is shallower. The simulation corresponds to Figs 10 and 11 in the main text.
(AVI)

S8 Movie Model of border cell cluster under influence of TIM force corresponding Figs 10 and 11 in the main text. (AVI)
