# Supplementary material for: Phase-field modeling of border cell cluster migration in Drosophila
Source: PLoS Comput Biol. 2026 Apr 22;22(4):e1014176. doi: 10.1371/journal.pcbi.1014176 (PMC13128136; doi:10.1371/journal.pcbi.1014176)
Supplement: S1 Text — (PDF) [file pcbi.1014176.s001.pdf]

1 Supplementary Document

1.1 Impact of Extracellular Geometry on Chemoattractant Distribution and Force Dynamics

To understand how extracellular tissue architecture affects border cell migration, we investigate the impact of domain geometry on steady-state chemoattractant concentration and the resulting migration forces. In particular, we compare two modeling approaches: one that assumes uniform extracellular geometry (constant cross-sectional area), and another that incorporates spatial variation in extracellular space by modeling a position-dependent cross-sectional area along the anterior-posterior (A–P) axis of the egg chamber.

Our goal is to evaluate how these two chemoattractant profiles influence the behavior of the chemotactic force (F_{chem}) and the tangential interface migration force (F_{TIM}). Because F_{chem} depends directly on spatial gradients in chemoattractant concentration, it is sensitive to local variations introduced by domain geometry. In contrast, F_{TIM} arises from interfacial mechanical interactions and is indirectly affected through changes in cluster position and geometry rather than the gradient itself. By analyzing migration patterns under each concentration model, we demonstrate that geometrically realistic extracellular domains can significantly weaken chemotactic cues, leading to slower or more erratic movement under F_{chem}, while F_{TIM} remains effective in maintaining directed migration.

By assuming a positive cross-sectional area $A(x) > 0$, we obtain the strong form of the equation:

$$\begin{aligned} \frac{\partial c}{\partial t} &= \frac{1}{A}\frac{\partial}{\partial x}\left(DA(x)\frac{\partial c}{\partial x}\right) - kc, \\ -DA(x)\frac{\partial c}{\partial x}|_{x=L} &= -\sigma, \\ -DA(x)\frac{\partial c}{\partial x}|_{x=0} &= 0, \end{aligned} \tag{A}$$

where L is the length of the egg chamber, and the boundary conditions reflect no flux at $x = 0$ and secretion at $x = L$. The steady state solution of Eq. (A) is shown in Figure 4(a).

By considering $A(x) \equiv 1$, in Eq. (A), the steady-state solution of this equation with boundary conditions is given by

$$c^*(x) = \frac{\sigma}{D\sqrt{\frac{k}{D}}\sinh(\sqrt{\frac{k}{D}}L)}\cosh\left(\sqrt{\frac{k}{D}}x\right). \tag{B}$$

We extend the steady-state solution $c^*(x)$ uniformly along the lateral y-direction. Specifically, we define the 2D concentration field as $c(x, y) = c^*(x)$, assuming no variation in the y-direction, Figure A(a).

We apply the same functional form for the receptor-mediated response $\rho(c)$ as defined in Eq. (11) in the main text. Figure A(b) shows the receptor-mediated response function $\rho(c(x, y))$, which exhibits a slower rate of increase at both the anterior and posterior ends of the domain. In particular, the plateau near the posterior end (larger x) reflects saturation of receptor binding as the chemoattractant concentration approaches its maximum near the oocyte.

We apply the extended chemoattractant concentration field $c(x, y) = c^*(x)$ in the chemotactic force term F_{chem}, which is added to the right-hand side of the phase field evolution equation (Eq. (9) in main text) to simulate directed migration of the border cell cluster.

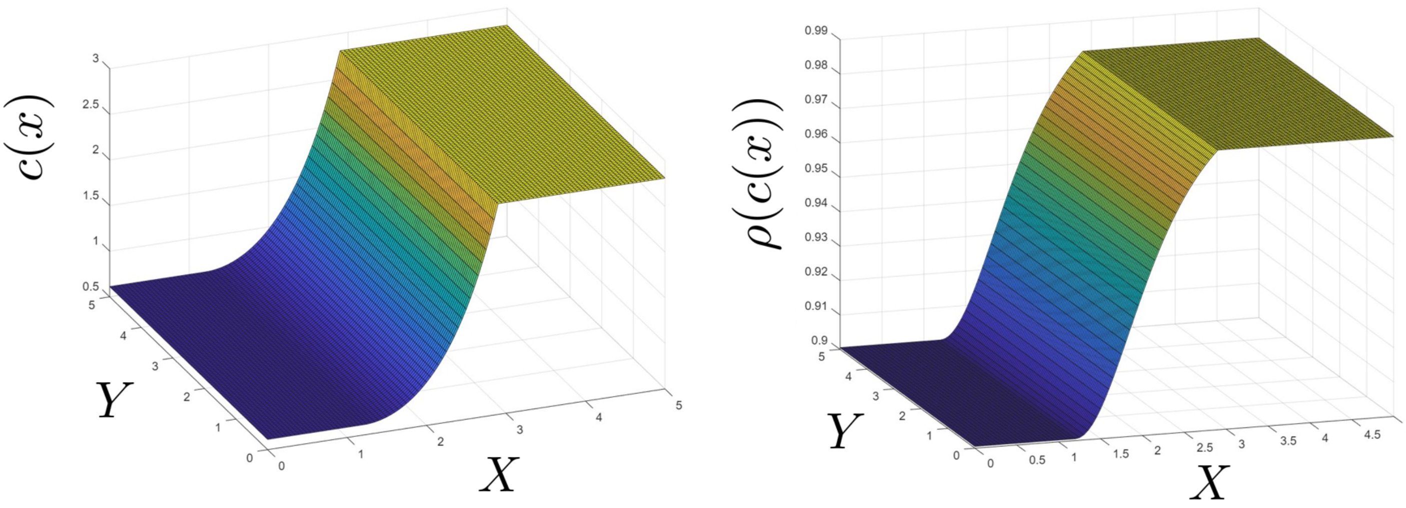

(a) (b)

Fig A. Steady-state chemoattractant concentration $c(x)$ and receptor response $\rho(c)$ in 2D. (a) The extended 2D chemoattractant concentration field $c(x, y) = c^*(x)$, where the steady-state solution $c^*(x)$ is derived from $A(x) \equiv 1$. (b) The response curve increases with x but shows slower growth near the anterior and posterior ends, particularly near the oocyte, where receptor saturation occurs due to high ligand concentration.

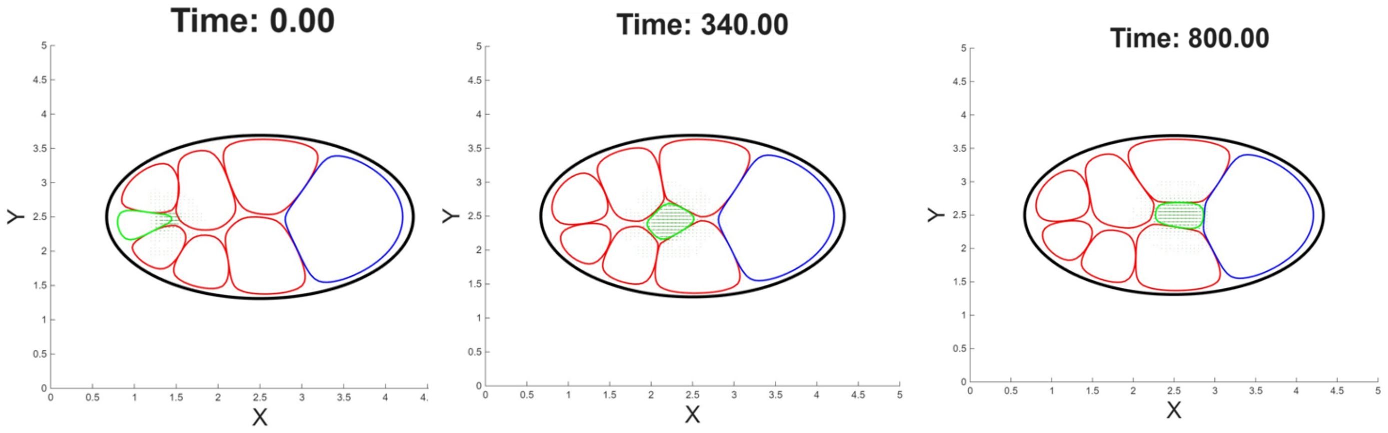

(a) (b) (c)

Fig B. Migration driven by F_{chem} chemotactic force using uniform chemoattractant concentration $c(x)$. Snapshots at three time points show the border cell cluster (green) migrating toward the oocyte (blue) under the influence of F_{chem}. The green vector field represents the gradient of the chemoattractant concentration ∇c, which increases in magnitude along the anterior-posterior axis, guiding forward movement.

In Figure B (a)-(c), the green vector field in each panel illustrates the direction and magnitude of the chemoattractant gradient ∇c, which determines the direction of F_{chem}. Due to the monotonic increase of concentration along the anterior-posterior axis, the gradient vectors consistently point toward the oocyte and gradually increase in magnitude along the migration path.

We compare the behavior of border cell migration under two different chemoattractant concentration models: $c(x)$, which assumes a uniform extracellular

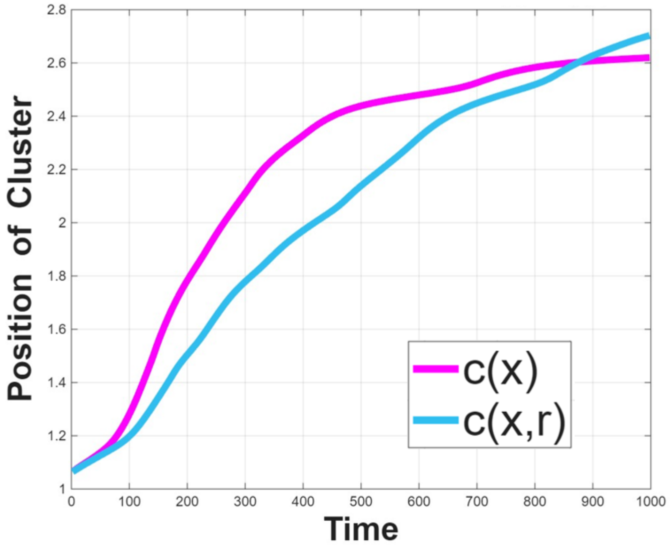

(a)

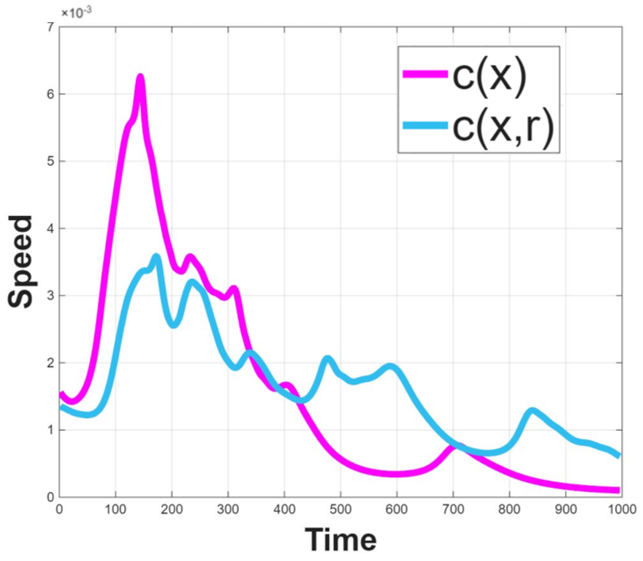

(b)

Fig C. Effect of extracellular space on F_{chem} chemotactic force–driven migration. (a) Position of the border cell cluster over time under two chemoattractant profiles: $c(x)$ (magenta), corresponding to a uniform extracellular domain with constant cross-sectional area $A \equiv 1$, and $c_r(x)$ (cyan), which accounts for spatial variation in extracellular geometry via a non-uniform area $A(x)$. (b) Corresponding cluster speed profiles. The non-uniform geometry $c_r(x)$ leads to reduced chemotactic gradients in certain regions, resulting in slower migration and more variable speed compared to the uniform case (cyan).

domain, and $c_r(x)$, which incorporates the influence of spatially varying extracellular geometry through a position-dependent cross-sectional area. The Figure C (a)-(b) shows how these differences affect both the position and speed of the migrating cluster.

To investigate how the Tangential Interface Migration (TIM) force responds to chemoattractant profiles independent of spatial heterogeneity, we simulate cluster migration using a uniform chemoattractant concentration based on $c(x)$ from Eq. (B) (Figure A), assuming constant cross sectional area.

In Figure D(a)-(c), we present snapshots of border cell cluster migration driven solely by the TIM force, as defined in Eq. (13) of the main text. The purple vector field illustrates the localized tangential interactions between the border cell cluster and adjacent nurse cells. These interactions generate contact-mediated traction that guides the cluster forward, even in the absence of spatial variation in chemoattractant concentration. To evaluate how extracellular space–dependent chemoattractant profiles influence TIM-driven migration, we compare the cluster's position and speed under uniform concentration $c(x)$ and spatially varying concentration $c_r(x)$, which accounts for changes in extracellular geometry.

To clarify the difference between F_{chem} and F_{TIM}, we work in a thin-interface viewpoint and ask how each force scales with the chemoattractant profile near the cluster. The chemotactic force is

$$F_{\text{chem}} = -\mu_c \nabla \cdot (\phi_c \nabla c),$$

which acts normal to the border-cell interface wherever ϕ_c varies. Projecting onto the local direction of the chemoattractant gradient $\hat{g} = \nabla c / |\nabla c|$ and using standard thin-interface expansions, one obtains an effective driving force

$$F_{\text{chem}} \cdot \hat{g} \;\approx\; \mu_c \, C_{\text{geom}} \, |\nabla c(x)|,$$

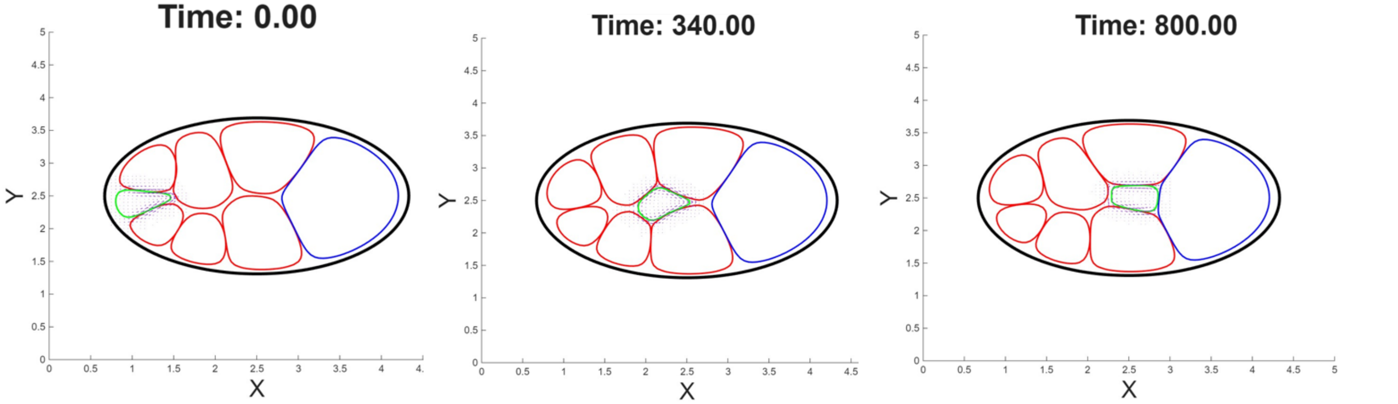

(a) (b) (c)

Fig D. Border cell cluster migration driven by TIM force under uniform chemoattractant concentration $c(x)$. Snapshots at three time points show migration driven by the tangential interface migration (TIM) force (Eq. (13) in main text). The purple vector field indicates tangential traction generated through contact between the border cell cluster (green) and adjacent nurse cells (red), enabling forward movement even in the absence of spatial heterogeneity in the chemoattractant field.

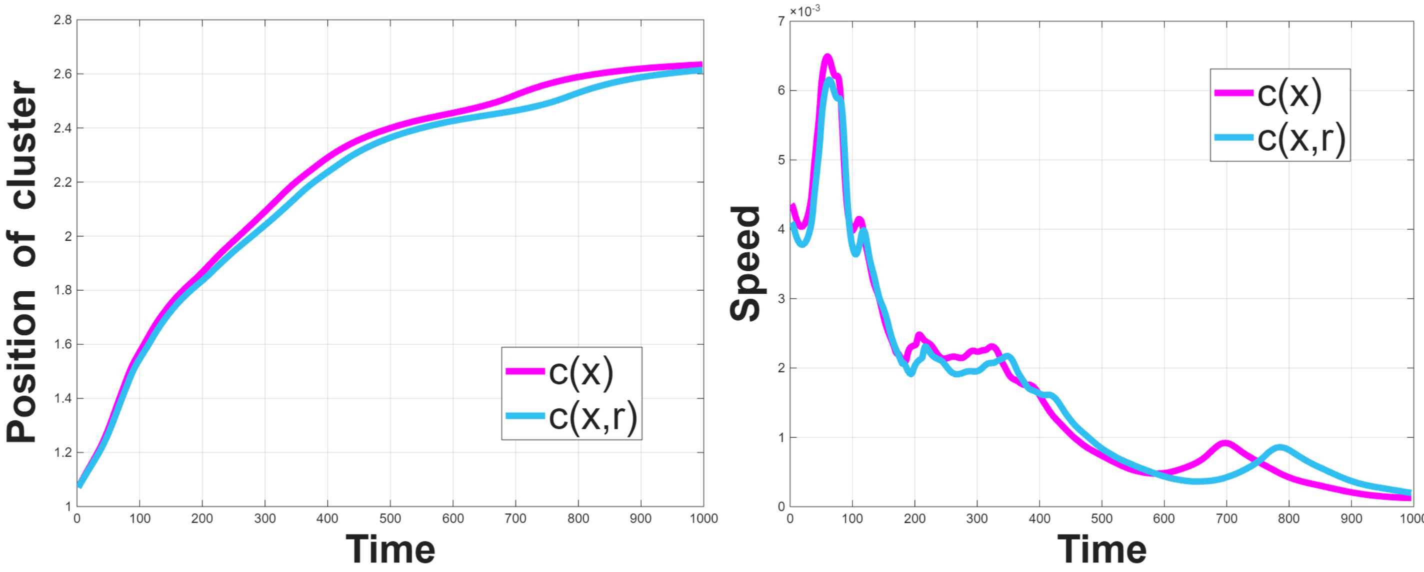

(a) (b)

Fig E. Comparison of TIM force–driven migration under uniform $c(x)$ and spatially varying $c_r(x)$ chemoattractant concentration. (a) Position of the border cell cluster over time for simulation using $c(x)$ (magenta) and $c_r(x)$ (cyan). (b) Corresponding cluster speed profiles. While overall migration remains robust under both conditions, subtle differences in speed and trajectory reflect the influence of chemoattractant field geometry on interfacial traction patterns driving the TIM force.

where x denotes the cluster position and C_{geom} is a positive constant that depends only on the interface geometry and the profile of ϕ_c across the interface. Thus the chemotactic propulsion scales linearly with the *magnitude* of the local chemoattractant gradient. In regions where $c(x)$ has negative concavity and the gradient flattens, $|\nabla c(x)|$ becomes small, and F_{chem} correspondingly vanishes. In this regime, chemotactic

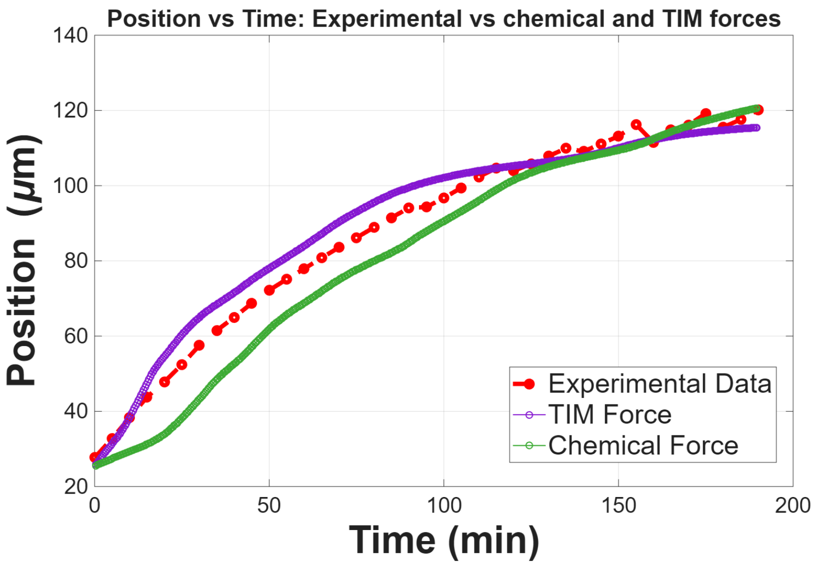

Fig F. Analysis of the live imaging data obtained for George et al [1], the average position of the border cell cluster (marked by slbo-lifeAct-GFP) by time over 13 live-imaging movies with images acquired every 5 minutes (red markers). Comparison of experimental border cell cluster position with model predictions under chemotactic and TIM forces over time as in Fig 8a.

guidance can stall the cluster even though the overall profile remains asymmetric.

The TIM force is given by

$$F_{\mathrm{TIM}} = -\bar{\mu}_c \nabla \cdot \Big(\rho(c)\, \phi_c \phi_j \; \mathrm{sgn}(\nabla c \cdot \nabla \phi_c^{\perp})\, \nabla \phi_c^{\perp} \Big),$$

where $\nabla \phi_c^{\perp}$ is oriented tangentially along the border cell nurse cell interface. In the same thin interface limit, the dominant contribution comes from a narrow layer around the interface Γ where $|\nabla \phi_c|$ is large. Projecting again onto $\hat{g}$ and integrating across the interface, one obtains an effective tangential driving term of the form

$$F_{\mathrm{TIM}} \cdot \hat{g} \;\approx\; \bar{\mu}_c\, \rho\big(c(x)\big)\, C_{\mathrm{TIM}}\; \mathrm{sgn}\,\big(\nabla c(x) \cdot \tau(x)\big),$$

where $C_{\mathrm{TIM}} > 0$ collects geometric factors (including the integral of $|\nabla \phi_c|^2$ along Γ) and τ is the local unit tangent along the exposed interface. Importantly, in this approximation the magnitude of the TIM driving is set by $\rho(c)$ and the interface geometry and depends only on the "sign" of the projected gradient through $\mathrm{sgn}(\nabla c \cdot \tau)$. As long as the chemoattractant field varies monotonically across the footprint of the cluster, the projected gradient maintains a well-defined sign, and the TIM-generated traction remains $\mathcal{O}(1)$ even when $|\nabla c|$ is small.

Together, these scalings explain the different sensitivities to concavity. For purely chemotactic guidance, $F_{\mathrm{chem}} \propto |\nabla c|$ and the driving force collapses in broad, weakly sloping or concave regions of the profile, leading to stalling. In contrast, TIM aggregates tangential receptor activation along the interface and depends on the sign rather than the magnitude of the projected gradient, so that the cluster can continue to migrate through shallow or concave regions until it reaches a true extremum where the gradient changes sign across the interface.

Parameter	Description	Value/Equation
ϕ	Phase field variable indicating cell presence	Eq. (1)-(10)
ϵ	Interface width control in Allen–Cahn equation.	Eq. (1)
$g(\phi)$	Double-well energy function for each cell phase ϕ_m	Eq. (1)
$V(t)$	Volume functional over phase field domain	Eq. (2)
$h(\phi)$	Smooth interpolation function localizing interface region	Eq. (3)
$\bar{V}(t)$	Volume functional over phase field domain	Eq. (1)
$\rho(c)$	Receptor activation level	Eq. (11)
E_i $(0 \leq i \leq 3)$	Components of total energy: territory, volume, adhesion.	Eqs. (1)-(6)
σ	Chemoattractant secretion rate.	Eq. (A)
A	cross-sectional area	Eq. (A)
k	Degradation rate of chemoattractant.	Eq. (A)
L	Length of egg chamber	Eq. (A)
ϵ_m^2	Interface width parameter for phase field ϕ_m.	[0.001, 0.001, 0.0005]
α_0	Energy intensity for cells	100
α_m	Strength of volume constraint for cell m.	100
β_0	Intensity of domain territories (epithelial-cells)	0.9
β_m	Intensity of domain territories (cell-cell)	$\beta(1,1) = \beta(1,2) = \beta(2,1) = 0.25$<br>$\beta(1,3) = \beta(3,1) = 0.25, \beta(2,3) = \beta(3,2) = 0.3$<br>$\beta(2,2) = \beta(3,3) = 0$
γ_0	Intensity of adhesion force (epithelial-cells)	0.007
γ_m	Intensity of adhesion force (cell-cell)	$\gamma(1,1) = 0.003, \gamma(1,2) = \gamma(2,1) = 0.004,$<br>$\gamma(1,3) = \gamma(3,1) = 0.008, \gamma(2,3) = \gamma(3,2) = 0.005$<br>$\gamma(2,2) = \gamma(3,3) = 0$
$c_0 = c(x,0)$	Initial chemoattractant concentration profile.	0
Δt	Time step for numerical simulation.	0.05
h	Size of spatial grid	0.05
M	Number of grid points in each spatial direction.	$5/0.05 + 1$
μ	Mobility parameter in phase field evolution.	0.025
μ_c	Mobility parameter in chemical force Eq. (12).	0.045
Γ	control the sensitivity	0.01
s	maximal activation level of receptor	1
ℓ	control the sensitivity	0.05
$\bar{\mu}_c$	Strength of TIM force	—
Ω	Two-dimensional square computational domain.	$[0,5] \times [0,5]$
D	Diffusion coefficient	1

Table A. Description of parameters of the phase field model.

References

1. George A, Akhavan N, Peercy BE, Starz-Gaiano M. Chemotaxis of Drosophila Border Cells is Modulated by Tissue Geometry Through Dispersion of Chemoattractants. iScience. 2025;.
